# Atypical experiences of captive chimpanzees (*Pan troglodytes*) are associated with higher hair cortisol concentrations as adults

**DOI:** 10.1098/rsos.170932

**Published:** 2017-12-13

**Authors:** S. L. Jacobson, H. D. Freeman, R. M. Santymire, S. R. Ross

**Affiliations:** 1 Lester E. Fisher Center for the Study and Conservation of Apes, Lincoln Park Zoo, Chicago, IL, USA; 2 The Davee Center for Epidemiology and Endocrinology, Lincoln Park Zoo, Chicago, IL, USA

**Keywords:** chimpanzee, cortisol, animal welfare, human–animal interaction, atypical rearing, development

## Abstract

Experiences during early development are influential on the lives of human and non-human primates into adulthood. The population of captive chimpanzees in the USA can provide insight into this relationship, as collectively they have experienced a wide range of exposure to both conspecifics (those raised in natal groups) and humans (those raised as personal pets or performers). Our study investigated chimpanzee exposure to humans using a continuous measure of categorization, the chimpanzee–human interaction index, and the relationship between this experience and cortisol concentrations in adulthood. Historical records and hair samples were collected from 60 chimpanzees which were socially housed in 13 zoos and sanctuaries. We found that more human exposure throughout the life of a chimpanzee was associated with higher hair cortisol concentrations in adulthood. Sex was also a significant factor affecting cortisol concentration, with male chimpanzees having higher cortisol concentrations than female chimpanzees. These results build upon the extensive literature about aversive effects of atypical social histories for chimpanzees and emphasize to managers the importance of monitoring potential negative health consequences and social deficits these individuals may exhibit.

## 1. Introduction

Experiences during infancy and the juvenile stage are particularly influential on the lives of human and non-human primates due to their prolonged developmental periods before reaching sexual maturity [1,2]. Primates with a typical developmental experience spend infancy in direct contact with their mother and other conspecifics, both kin and non-kin. This period of dependence on their mother as their source of food and protection provides opportunities to learn species-typical behaviours through observation, physical contact and communication, which contribute to reproductive and social competence as adults. For many primates, these opportunities for learning continue through juvenility as they become more independent and begin to interact with others of their own species [3].

The importance of a primate's social environment is underscored by the cognitive and behavioural deficits demonstrated by primates, human and non-human, raised in socially deprived environments [4,5]. Given that the primary early social relationship for many primates is with their mothers, the influence of maternal care has been broadly studied, including the effects of an absence of maternal care. Captive non-human primate models are often used to investigate the importance of the maternal relationship along a spectrum from complete isolation (e.g. [5]) to repeated maternal separations (e.g. [6]) and peer-rearing (reviewed by [7]). Such studies have demonstrated that non-human primates reared with decreased or absent maternal care exhibit elevated abnormal behaviour that generally persists into adulthood (e.g. macaques [8]; chimpanzees [9]).

While maternal care is clearly important, there are other important social experiences that are influential throughout a primate's life, particularly for a gregarious primate species. Many primates live in large social groups in the wild and spend a significant portion of time with individuals other than their mother [10]. In social groups, interactions with conspecifics, such as play, help these primates learn appropriate social behaviours which in turn may aid in social integration [11]. In one study of macaques reared only with other infants, they developed social behaviours associated with grooming, aggression, play and sex, although some of these behaviours appeared to be developmentally delayed compared with typical maternal rearing [12]. The degree of conspecific interaction also appears to have long-term effects, as macaques who were not reared by their mothers, but experienced more conspecific interaction than another group with limited social interaction, had higher social ranks as adults [13]. For some primates, social interaction with conspecifics appears to buffer against the development of abnormal behaviours that may occur with insufficient maternal exposure. In a study of captive chimpanzees, those who lived in groups with peers for part of their developmental period exhibited less abnormal behaviour than those raised only by humans, and surprisingly even less than those only raised by their mothers [14]. Therefore, it is evident that social interactions with conspecifics also contribute significantly to normal social and cognitive development.

Unlike their wild counterparts, captive primates are influenced by social interactions not only with members of their own species but with humans as well, including formal and informal interactions with caregivers and unfamiliar humans such as visitors to the facilities. These experiences can include potentially intensive human exposure through hand rearing during infancy, which has been connected with the development of abnormal behaviours in several species [8,10]. Frequent and intensive interactions with humans have also resulted in enculturation effects in which apes displayed different social skills and cognitive performance from those with reduced human influence [15,16]. Less intense interactions between humans and captive chimpanzees can also affect chimpanzee behaviour. The increased presence of humans seemed to heighten aggression and wounding in laboratory-housed chimpanzees [17] and even casual and positively intended interactions with human caretakers can influence the behaviour of zoo-housed chimpanzees in some cases [18].

The population of chimpanzees in the USA has experienced a variety of human interactions, as these chimpanzees have lived in a wide range of settings, including zoological parks, research laboratories and sanctuaries, as well as in private ownership as pets or ‘actors' for various forms of media. This diverse population has collectively experienced a broad array of rearing conditions and variable exposure to conspecifics and humans depending on their residency and use by humans. Some chimpanzees have experienced species-typical development with maternal rearing in a group of conspecifics, while others had less typical upbringings, including little to no maternal interaction and high degrees of exposure to humans early in life (best evidenced by those raised as pets and performers). Such variation provides a unique opportunity to investigate the long-term effects of experiences with conspecifics and humans.

A set of previous studies leveraged this population variability to investigate the relationship between the degree of early typical and atypical social exposure and adult outcomes for this species. Freeman & Ross [19] examined adult behaviour of 60 chimpanzees and found significantly higher rates of social grooming in chimpanzees with more exposure to conspecifics during infancy than those with mixed or mostly human exposure. Freeman *et al.* [20] also investigated the relationship between the developmental continuum of social exposure and personality traits in this chimpanzee population. Those chimpanzees who had spent less time with conspecifics as infants were rated as less extraverted as adults than those who had more human exposure as infants.

These studies suggest atypical developmental periods and later experiences characterized by high degrees of human interaction result in long-term social deficits for chimpanzees, but is this change also reflected by physiological measures of chimpanzees' welfare? A common means of evaluating welfare is measuring stress through the activity of the hypothalamic-pituitary–adrenal (HPA) axis and subsequent levels of the cortisol hormone. The HPA axis increases in activity in response to stress which results in elevated concentrations of glucocorticoids, including cortisol. This endocrine response is adaptive, as it increases the immediate availability of energy, partially by inhibiting certain physiological processes that may not immediately affect survival. If the stressor continues or stress response is dysregulated, glucocorticoid levels can remain high and have deleterious effects, such as suppressing immune function and reproduction for long periods of time [21]. Social relationships may further influence these cortisol levels as there is evidence that subordinate social status can result in higher cortisol levels in several species [22] and that affiliative social interactions are associated with decreased cortisol levels in primates [23].

Long-term physiological effects of early life histories have been investigated in a variety of ways in past primate studies, primarily relating to atypical rearing. Using cortisol concentrations in both blood and hair samples, researchers have uncovered variable results when comparing mother rearing to other rearing exposures [8,13,24,25]. A study by Yamanashi *et al.* [26] used hair cortisol concentrations to assess the effect of various rearing backgrounds. Unexpectedly, they found that chimpanzees separated from their mother after 1 year had lower cortisol concentrations than any other group. A different study reported that chimpanzees who were separated in their first year had a greater cortisol response to relocation than those separated after a year with their mother [27]. These results further emphasize the importance of early life histories on captive chimpanzees and the ability to examine long-term effects of a range of experiences using physiological measures.

Since sociality is an important part of chimpanzee life, we aimed to investigate whether the social differences would be reflected physiologically by differences in cortisol levels between chimpanzees with differing degrees of conspecific exposure throughout their lives. To accomplish this, we used a continuous measure of chimpanzee and human exposure, the chimpanzee–human interaction index (CHI) used in previous studies and described in detail in Freeman & Ross [19], to characterize the experiences of these chimpanzees. We analysed cortisol levels from the chimpanzees' hair, which represents several months of cortisol accumulation and therefore provides a good representation of baseline levels of HPA activity [28]. We predicted that chimpanzees with less conspecific exposure over their lifespan would have higher cortisol levels later in life. The results of these analysis have potential application for chimpanzee caretakers in their planning of social introductions and group management, and may inform strategies to accommodate individuals who may be prone to more stress due to social histories.

## 2. Methods

This study complied with protocols approved by the Chimpanzee Species Survival Plan (SSP) management group and animal care committees at each participating institution.

## 3. Subjects

The subjects of this study were 60 chimpanzees (24 males; 36 females) with a range of experience with other chimpanzees and humans. None of the chimpanzees were wild born and none had lived in laboratories. The chimpanzees lived in 13 different zoos and sanctuaries. They ranged in age from 10 to 54 (mean = 24 years).

Subjects were evaluated in their current housing at sanctuaries accredited by the Global Federation of Animal Sanctuaries (Center for Great Apes and Save the Chimps) or zoological parks accredited by the Association of Zoos and Aquariums (Dallas Zoo, Henry Vilas Zoo, Houston Zoo, Zoo Knoxville, Little Rock Zoo, North Carolina Zoo, Oakland Zoo, Oklahoma City Zoo, Oregon Zoo, Sunset Zoo and Tulsa Zoo). Fifty-nine of the subjects were socially housed. The one individually housed chimpanzee was kept as a solitary pet for over 30 years and attempts to introduce her to conspecifics had not been successful at the time of the study.

## 4. Chimpanzee–human interaction index

The CHI was a novel index developed by Freeman & Ross [19]. Management and historical records from institutions holding the chimpanzees were used to determine the degree that each individual was exposed to humans and/or chimpanzees throughout their life. For each day of their life, a numerical value was determined based on three categories of exposure: full exposure to conspecifics, full exposure to humans, and mixed exposure to both conspecifics and humans. For example, a chimpanzee that lived in a social group in a zoo would have a proportion of 1/1 for the day because they spent 100% of their time with conspecifics. A pet chimpanzee raised with a human family without exposure to other conspecifics, would have a proportion of 0/1 for the day. A performing chimpanzee with relatively equal exposure to small groups of conspecifics and human trainers and audiences would have a proportion of 0.5/1. The CHI value for the lifetime of a chimpanzee would be the sum of the scores divided by the total possible score, the total number of days they had been alive.

## 5. Hair collection

Caregivers collected one to four samples from each chimpanzee over a one-year period. There were at least three months in between each hair collection. Hair samples were collected from the same place on the chimpanzee depending on the individual and what was feasible for the institution, but was consistent for each subject. Hair was collected from the chimpanzees from the base of the skin using scissors, rather than pulling the hair to ensure that the follicles were not included in the sample. The samples were 3–5 cm in length and were stored at room temperature in foil.

## 6. Cortisol enzyme immunoassay

Hair cortisol analysis methods were based on the protocol developed by Davenport *et al*. [29]. Hair was placed into pre-labelled 16 × 125 mm plastic tubes. Five millilitres of 90% methanol was added to the tubes and they were capped and vortexed gently for 30 s. The excess methanol was then poured into a container to be air dried. This procedure was repeated for a total of three washes. The hair was then placed in pre-labelled weigh boats and allowed to dry for 3–5 days under a hood, before cutting it into small 2–3 mm sections. Any portions containing the hair follicle were discarded. The hair was placed into pulverizing vials with tweezers which were washed with isopropanol in between each sample. The hair was pulverized using an Omni Bead Ruptor 24 with 2 ml tubes with metal beads. The machine was run at 6.8 m s^−1^, 50 s × 2, with a 15 s break. The hair was then removed from the vials and placed into aluminium weigh boats. Pulverized hair was weighed out in increments of 0.02 ± 0.005 g and placed into pre-labelled 16 × 125 mm plastic tubes. Two millilitres of 90% methanol was added to the tubes and they were briefly vortexed. The tubes were then placed on a Glas-col mixer for four hours at a speed of 50. The tubes were then centrifuged for 15 min at 1500 r.p.m. The extracts were poured off into a second set of labelled 12 × 75 mm plastic tubes and the tubes were dried under air. Three to five glass beads were added to each tube and the dried extract was reconstituted in 500 µl of dilution buffer. The tubes were then vortexed briefly and sonicated for 20 min.

The cortisol was assayed using a cortisol enzyme immunoassay. Cortisol polyclonal antiserum and horseradish peroxidase (R4866; provided by C. Munro, Davis, CA, USA) were used at a 1 : 8500 and 1 : 20 000 dilution, respectively. Cross-reactivity to the cortisol antiserum was analysed as described in [30]. The cortisol enzyme immunoassay was validated by demonstrating: (i) parallelism between binding inhibition curves of hair extract dilutions (4*x − *1 : 2; *r* = 0.973) and (ii) significant recovery (greater than 90%) of exogenous cortisol added to hair extracts (1 : 200; $\hat{y}=y=0.9146x+7.5354$; *R*² = 0.9917; *p* < 0.001). Intra-assay variability was less than 10% CV and inter-assay variability was less than 20% CV.

## 7. Analysis

Analyses were conducted in R v. 3.2.3 [31]. Hair cortisol concentrations were normalized using a log_10_ transformation (see electronic supplementary, table S1, for average cortisol concentrations and ranges for each chimpanzee before log transformation). Pearson's correlations were initially used to determine a relationship between CHI and the average log_10_ cortisol concentration. A linear mixed effects analysis was performed to analyse the relationship between CHI and log_10_ cortisol concentrations using the lme4 package in R [32]. CHI and sex of the subjects were included as fixed effects while the current institution housing the subjects and the subject ID were included as random effects. Likelihood ratio tests using the ANOVA function and *χ*^2^ distribution were used to compare the null model including sex, subject and institution to a full model containing those factors and CHI. The assumptions of linearity and the absence of heteroskedascticity were examined through plotting residuals and the assumption of normality was inspected through visualization of a Q–Q plot. There was also no evidence of collinearity between predictor variables.

## 8. Results

**Figure 1.**
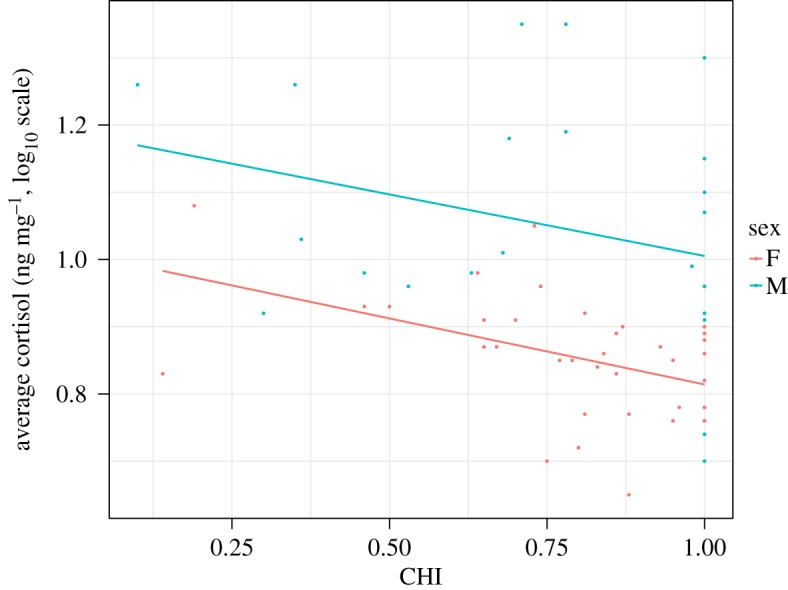
Correlation between CHI and average log_10_ cortisol concentrations for each subject. A CHI of 0.0 is high human exposure and 1.0 is high chimpanzee exposure.

CHI of the subjects ranged from 0.1 to 1.0, where 0 was full human exposure and 1 was full chimpanzee exposure. The average CHI was 0.78 (s.e.m. = 0.03). The average log_10_ cortisol concentrations for each subject ranged from 0.65 to 1.35 ng mg^−1^, and the average across subjects was 0.93 ng mg^−1^ (s.e.m. = 0.02). Average log_10_ cortisol concentrations were negatively correlated with CHI (*r* = −0.32, *p* < 0.05) such that the more conspecific exposure that chimpanzees experienced, the lower the average cortisol concentrations (figure 1).

The full regression model including CHI was significantly different from the null model that omitted it (*χ*^2^(1) = 4.89, *p* = 0.027). CHI predicted log_10_ cortisol concentration, with a higher index (more chimpanzee exposure) being associated with lower cortisol concentrations. Sex was also significant in the full model, with males showing log_10_ cortisol concentrations increased by 0.173 ng mg^−1^ (s.e. = 0.03 ng mg^−1^) over females. However, the interaction between CHI and sex was not significant when models were compared with and without the interaction effect (*χ*^2^(1) = 0.07, *p* = 0.79). Complete model results are reported in tables 1 and 2.

**Table 1.** Results of the full linear mixed model testing the influence of CHI, subject, institution and sex on adult log_10_ cortisol concentrations.

random effects	variance	s.d.	
subject (intercept)	6.138 × 10^−3^	7.835 × 10^−2^	
institution (intercept)	1.100 × 10^−16^	1.049 × 10^−8^	
residual	2.032 × 10^−2^	1.425 × 10^−1^	

fixed effects	estimate	s.e.	*t* value
intercept	0.99417	0.05306	18.735
CHI	−0.17308	0.06235	−2.776
sex (male)	0.17137	0.03010	5.694

**Table 2.** Results of the null linear mixed model testing the influence of subject, institution and sex on adult log_10_ cortisol concentrations.

random effects	variance	s.d.	
subject (intercept)	0.005400	0.07348	
institution (intercept)	0.002463	0.04963	
residual	0.020353	0.14266	

fixed effects	estimate	s.e.	*t* value
intercept	0.83952	0.02394	35.07
sex (male)	0.19022	0.03009	6.32

## 9. Discussion

We found that more human exposure throughout chimpanzees' lives (lower CHI) was associated with hair cortisol concentrations later in life that were higher than those in chimpanzees with more conspecific exposure. The higher baseline cortisol levels of individuals with more human exposure indicate that their HPA axis is more active than the others. Similar to our results, exposure to stress in the early lives of humans and rats has also been linked to hyperactivity of the stress response [33,34], but see also [35,36]. If the HPA axis activity is prolonged it can detrimentally suppress an individual's immune response, affect brain function and reproduction as described in several species [21,24,37]. These results support anecdotal evidence from sanctuaries that the circumstances experienced in a chimpanzee's previous life experience can affect their stress levels even many years later as adults. The effects of these early experiences can be expressed also in particular circumstances. Previous research demonstrated that maternal deprivation can impact chimpanzees' ability to cope with stressful events such as relocation [26,27]. Our study reinforces such results and also serves to show that these effects are demonstrable even outside acutely stressful circumstances. The two previous studies with this population [19,20] illustrate that chimpanzees with high levels of human exposure tend to be characterized by lower prosocial behaviour and less extraverted personalities and these ‘social handicaps’ may play a role in explaining the elevated hormonal profiles of these chimpanzees.

Traditionally, studies examining the effects of primate rearing experience have used categorical designations for rearing: mother-reared, peer-reared, human-reared, etc. Despite the convenience of these designations, these categories fail to accurately characterize the more nuanced spectrum of experiences that exist for captive primates that are likely influenced by both conspecifics and their human caretakers. The population under study here represents chimpanzees with highly atypical histories with very little exposure to conspecifics (pets, performers), those raised in their natal groups with high exposure to conspecifics in large zoo environments, and those with a mix of exposures over their developmental period. Our use of a continuous measure of human exposure, the CHI, therefore makes this study unique in characterizing adult physiological stress based on a more flexible measure of social exposure than previous studies.

Considering the atypical early experience of many pet and performer chimpanzees, it is not surprising that these physiological aberrations develop. Chimpanzees bred for the private pet trade are typically removed from their biological mothers very early in their life (often within the first few days) in order to promote tractability for human interaction. They are sold to private owners and typically raised like humans and exposed to human social behaviour and environments. They tend to have very little exposure to other chimpanzees until they grow too large and dangerous to manage, at which time they are transferred to other locations, including sanctuaries and zoos, where they may have their first interactions with other chimpanzees. Performer chimpanzees typically follow a similar developmental trajectory, with early removal from the mother and high exposure to humans, familiar and unfamiliar, early in life as they are trained to perform atypical behaviours for audiences. They may, however, have some limited contact with other performer chimpanzees being managed in the facility, although these conspecifics also lack normal socialization and may behave atypically. Both types of early life experience lack the natural opportunity for chimpanzees to learn species-typical responses to social behaviours such as play or grooming, as demonstrated by the previous study with this population [19].

Without this social knowledge, it is possible that chimpanzees with high degrees of human exposure struggle to integrate into chimpanzee social groups, leading to the higher level of stress identified in our sample, compared to individuals who had the chance to develop appropriate social skills. Kalcher-Sommersguter *et al*. [38] demonstrated that chimpanzees who had atypical early social histories had impaired social integration later in life, particularly in their grooming networks. Grooming is critically important for chimpanzees in navigating their social environment, as it is used to strengthen social bonds and reconcile conflict (e.g. [39]). Other studies investigating conflict in differently reared chimpanzees found that individuals who experienced atypical rearing demonstrated less coalitionary behaviour than those who were mother-reared [40]. Chimpanzees who did not experience mother-rearing also suffered more wounding than those who did; an indicator that they may not have been well equipped to resolve social conflict in some cases [41]. Therefore, less conspecific experience could generally impair navigation of social conflict, leading to stress that is not resolved as part of these interactions.

Our results also demonstrated that sex was associated with hair cortisol concentration, with males exhibiting higher cortisol concentrations than females. Ours is not the first to find such a sex difference in hair cortisol [26], though this effect is not consistently reported [42]. Yamanashi *et al*. [26] also found that male cortisol concentrations were positively associated with receiving high levels of aggression, suggesting that male chimpanzees may be more reactive than females to stressors such as targeted aggression. This heightened sensitivity of male chimpanzees has also been suggested by studies that have found higher rates of abnormal behaviour in males than in females [43,44]. It is also possible that differences in metabolic stress between the sexes may also account for variation in cortisol concentrations, especially due to dominance displays by male chimpanzees [45]. The relationship between sex and cortisol requires further investigation to augment our understanding of these findings and to inform managers when considering chimpanzee relocations and introductions.

While this study leverages a relatively large sample of chimpanzees and data drawn from decades of records, there are limitations that must be considered in interpreting the findings. An important limitation of the CHI is that it does not account for subtle but possibly influential differences in relationship quality which likely varies between individuals and may influence social development. It is also important to consider that our analysis looked at an individual's CHI value over their lifetime, but atypical experiences in a chimpanzee's first years of life could have a more significant effect on an individual because this time period is particularly influential for socio-cognitive development [46]. We also acknowledge that factors during development other than social histories may influence an individual's stress response later in life. For instance, stress responses may vary due to individual genetic differences, which were not analysed in this study. Even with these limitations, our study provides important evidence suggesting a relationship between early social development of chimpanzees and their adult stress levels. While we cannot conclusively demonstrate causality in the relationship, the most parsimonious explanation is that the higher cortisol expressed by the low CHI chimpanzees were the result of those atypical social interactions.

There is already substantial literature documenting the range, both in form and severity, of effects that result from non-maternal rearing in primates [47,48]. This study, along with the earlier studies on this population of chimpanzees [19,20] builds on that extant knowledge in a very specific way: by recognizing and leveraging the inherent continuous nature of human/conspecific social influences that may affect young captive chimpanzees, and by demonstrating that the long-term outcomes of atypical social environments have the potential to affect individual development in multiple ways including personality, behaviour and physiology. While these outcomes are likely to be intertwined, the consistent nature of these multiple measures further emphasizes the substantive influence of being reared by those of another species.

These findings fit well with what is already known about the importance of the early species-typical social environments for primates and also have potential applications for captive management. This evidence serves to reinforce best practices for animal managers, who already strongly advocate for maternal rearing whenever possible [49]. Furthermore, the knowledge that chimpanzees with high exposure to humans during early development can express different and perhaps challenging social profiles as adults, may aid managers in selecting social groups and adapting group management strategies. Despite the challenges these chimpanzees face in terms of their socio-behavioural development, careful management at several zoos and sanctuaries has demonstrated that even these individuals can successfully be integrated into functioning social groups and reap the array of benefits of appropriate social companionship. Finally, although the prevalence of chimpanzees in pet and performing circumstances is on a stark downward trajectory due to recent legislative and regulatory change, these data bolster the evidence that such practices can incur significant and long-term harms for chimpanzees, and may serve to further discourage such activities.

## Supplementary Material

Table of Averages and Ranges of Chimpanzee Cortisol Concentrations

## Data accessibility

The datasets supporting this article have been uploaded as part of the electronic supplementary material and raw data are available from the Dryad Digital Repository at https://doi.org/10.5061/dryad.538jd 50].
