# Supplementary material for: Atypical experiences of captive chimpanzees (Pan troglodytes) are associated with higher hair cortisol concentrations as adults
Source: R Soc Open Sci. 2017 Dec 13;4(12):170932. doi: 10.1098/rsos.170932 (PMC5750001; doi:10.1098/rsos.170932)
Supplement: Table of Averages and Ranges of Chimpanzee Cortisol Concentrations [file rsos170932supp1.docx]

Supplementary Table 1. The average hair cortisol concentrations for each chimpanzee and the range between multiple hair samples. A “NA” as the range value indicates that there was only one hair sample provided for that chimpanzee.

| **Chimpanzee ID** | **Average cortisol concentration (ng/mg)** | **Range of cortisol concentrations (ng/mg)** |  | **Chimpanzee ID** | **Average cortisol concentration (ng/mg)** | **Range of cortisol concentrations (ng/mg)** |
| --- | --- | --- | --- | --- | --- | --- |
| 001 | 5.96 | 2.35 |  | 031 | 12.92 | 8.65 |
| 002 | 7.35 | 2.59 |  | 032 | 9.71 | 4.92 |
| 003 | 8.06 | 9.02 |  | 033 | 5.32 | 3.63 |
| 004 | 4.50 | NA |  | 034 | 7.68 | 2.36 |
| 005 | 9.02 | 9.62 |  | 035 | 8.22 | 6.43 |
| 006 | 9.85 | 5.26 |  | 036 | 8.71 | 6.61 |
| 007 | 7.14 | 2.82 |  | 037 | 5.75 | NA |
| 008 | 9.78 | 3.96 |  | 038 | 7.31 | 5.19 |
| 009 | 6.13 | 2.79 |  | 039 | 9.52 | 6.24 |
| 010 | 7.43 | 6.22 |  | 040 | 9.46 | 6.05 |
| 011 | 8.47 | 4.52 |  | 041 | 7.78 | 2.11 |
| 012 | 6.77 | 2.18 |  | 042 | 7.39 | 0.20 |
| 013 | 20.56 | 9.35 |  | 043 | 6.88 | 4.43 |
| 014 | 7.21 | 1.53 |  | 044 | 9.02 | 12.01 |
| 015 | 8.58 | 3.71 |  | 045 | 18.04 | NA |
| 016 | 23.21 | 14.81 |  | 046 | 8.27 | 2.40 |
| 017 | 8.66 | 3.17 |  | 047 | 11.94 | 2.46 |
| 018 | 8.15 | 12.65 |  | 048 | 5.55 | 2.26 |
| 019 | 7.99 | 2.45 |  | 049 | 21.28 | 25.91 |
| 020 | 12.28 | 5.58 |  | 050 | 9.22 | 2.54 |
| 021 | 10.36 | 4.06 |  | 051 | 9.66 | 2.77 |
| 022 | 5.33 | 2.21 |  | 052 | 5.22 | 3.44 |
| 023 | 10.61 | 0.64 |  | 053 | 7.78 | 1.25 |
| 024 | 5.74 | 1.59 |  | 054 | 6.75 | 2.03 |
| 025 | 7.72 | 0.62 |  | 055 | 15.43 | 8.46 |
| 026 | 6.13 | 3.07 |  | 056 | 5.92 | 1.03 |
| 027 | 16.66 | 11.70 |  | 057 | 11.37 | 3.65 |
| 028 | 16.39 | 25.48 |  | 058 | 7.56 | 4.59 |
| 029 | 22.66 | 10.64 |  | 059 | 9.98 | 10.76 |
| 030 | 8.13 | 0.59 |  | 060 | 5.70 | 4.14 |
